# The Mucous Membrane of the Uterus

**Published:** 1875-11

**Authors:** 


*The Mucous Membrane of the Uterus, with Special Reference to the Development and Structure of the Deciduæ.* By Geo. J. Engelmann, A. M., M. D.; Illustrated. New York: Wm. Wood & Co., 1875. Buffalo: H. H. Otis.

Dr. Engelmann's monograph first appeared in the American Journal of Obstetrics for May, 1875, from which it has been reprinted and presented, in a neatly bound form, to the profession.

The observations upon which this work are based were made in Vienna and Berlin, during the fall of 1871. In these investigations the author was assisted by Dr. Kundrat, first assistant to Prof. Rokitansky. During the course of the investigations over three hundred Uteri were examined, and the conditions of the mucous membrame examined in all its various stages from the time of its first appearance in the fœtus down to the period of involution and inaction.

The work is divided into three parts, viz:—I. The Mucous Membrane of the Womb, in its development up to the time of Puberty. II. The Mucous Membrane during its period of Maturity and Functional Activity, from the time of Puberty to the change of Life. III. The Mucous Membrane after the change of Life.

The first part is a careful and minute discription of the mucous membrane from fœtal life up to the period when it has arrived at functional activity. The author, from the various circumstances of nationality, mode of life, and health of the individual examined, is unable to determine any definite time at which this functional activity commences.

Dr. Engelmann does not receive the views of Pouchet, Tyler, Smith, and others, that the mucous membrane is shed at each menstrual period. Dr. John Williams, in a recent article in the *Obsterical Journal of Great Britain and Ireland*, gives the result of several observations upon this point, and his conclusions fully support the theory of Pouchet. The author of this work, in fact, does not seem very well settled in his opinion.

The author agrees with Coste and Dalton in the statement that menstruation and ovulation are coincident in point of time, but does not tell us how he will dispose of those cases in which pregnancy has occurred without menstruation; and also those in which a flow exactly resembling the menstrual flow has been observed for some months after the removal of both ovaries. The monograph shows a large amount of careful study of the numerous cases which the author had at his disposal, and although the conclusions reached in some instances conflict with long accepted views, the work will amply repay a careful reading.

